# Prognostic factors of acute and chronic graft-versus-host disease in pediatric patients undergoing bone marrow haploidentical transplant with post-transplantation cyclophosphamide

**DOI:** 10.1016/j.htct.2026.106539

**Published:** 2026-09-24

**Authors:** Meriem Lameri, Monia Ben Khaled, Eya Ghazouani, Ilhem Ben Fraj, Mohamed Hichem Sellami, Wafa Aissa, Yesmine Boughzala, Houda Kaabi, Slama Hmida, Monia Ouederni, Manel Chaabane

**Affiliations:** a Immunogenetics, Cell Therapy and Blood Transfusion Research Laboratory (LR20SP05), National Blood Transfusion Centre of Tunis, University of Tunis El Manar, Tunisia; b Pediatric Immunolo-Hematology Unit, Bone Marrow Transplantation Center of Tunisia, Tunis, Tunisia

**Keywords:** Hematopoietic stem cell transplantation, Haploidentical, Cyclophosphamide, Graft-versus-host disease

## Abstract

**Objective:**

Haploidentical hematopoietic stem cell transplantation has become an important therapeutic option for pediatric patients lacking an HLA-identical donor. This study aimed to identify clinical and transplant-related predictors of graft-versus-host disease in Tunisian children undergoing haploidentical hematopoietic stem cell transplantation with post-transplant cyclophosphamide.

**Methods:**

A retrospective study, conducted at the National Blood Transfusion Center, in collaboration with the National Bone Marrow Transplantation Center, included 47 pediatric patients transplanted between January 2019 and December 2023. Associations with acute and chronic graft-versus-host disease were assessed using contingency tables with odds ratios and 95% confidence intervals, followed by multivariate logistic regression for relevant variables.

**Results:**

Forty-eight transplant procedures were analyzed. In the univariate analysis, most parameters, including graft cellular content, conditioning regimen, donor age, underlying disease, donor-specific antibodies and ABO incompatibility, were not associated with graft-versus-host disease. In contrast, donor–recipient sex mismatch significantly increased the risk of acute graft-versus-host disease (OR = 10.0; 95% CI: 2.26–44.20; p = 0.0024) and was confirmed as an independent predictor on multivariate analysis (OR = 17.7; 95% CI: 0.13–297.9; p = 0.0068). No variable emerged as a significant determinant of chronic graft-versus-host disease.

**Conclusions:**

In this cohort, donor–recipient sex mismatch was the main risk factor for acute graft-versus-host disease, while no clear predictor was identified for chronic disease. Larger prospective studies are needed to validate these findings and to refine predictive models, with the ultimate aim of optimizing haploidentical hematopoietic stem cell transplantation protocols and improving outcomes.

## Introduction

It is well known that allogeneic hematopoietic stem cell transplantation (allo-HSCT) constitutes a major advance in the treatment of several malignant and non-malignant hematological pathologies [[1], [2], [3], [4]]. However, donor selection remains a crucial challenge, particularly in the absence of an HLA-matched donor. Therefore, haploidentical transplantation has emerged in recent years as a promising alternative [5,6].The administration of high-dose cyclophosphamide within days of transplantation has proven its efficacy in selectively eliminating alloreactive T lymphocytes, responsible for adverse immune reactions, while sparing regulatory cells and hematopoietic stem cells. This approach has transformed haploidentical transplantation into a viable and accessible strategy for a wider patient population. However, several prognostic factors still influence the outcome of this procedure, notably the occurrence of graft-versus-host disease (GVHD). Understanding these factors will not only help improve patient and donor selection, but also refine therapeutic strategies, with the ultimate aim of optimizing the chances of success and minimizing transplant-related complications [7,8].

In this study, the influence of key factors on the development of acute and chronic GVHD were investigated in a cohort of Tunisian pediatric recipients undergoing haploidentical bone marrow allo-HSCT. Patient-specific variables (such as underlying diagnosis and donor-specific antibodies), donor characteristics (including age, sex, and weight), graft composition (mononuclear and CD34^+^ cell content, and ABO compatibility) and procedural aspects (particularly the conditioning regimen) were assessed with the goal of better understanding how these factors shape transplant outcomes and ultimately improve patient care.

## Materials and methods

### Study population

This monocentric retrospective study was conducted at the Serology and Cell Therapy department of the National Blood Transfusion Center (NBTC) of Tunisia, in collaboration with the Pediatrics and Immunohematology department of the National Bone Marrow Transplantation Center (NBMTC). Forty-seven consecutive pediatric patients who underwent haploidentical bone marrow allo-HSCT between January 2019 and December 2023 were included. Clinical and biological data were collected from medical records.

### Graft characteristics

Bone marrow was harvested in the NBMTC operating room via posterior iliac crest puncture under general anesthesia. A maximum volume of 20 mL/kg of donor body weight was harvested and subsequently transported to the NBTC cell therapy unit. The bone marrow harvest was first filtered. Depending on the ABO compatibility between the donor and recipient, plasma depletion, red blood cell depletion, or both were performed. Mononuclear cell (MNC) counts of bone marrow grafts were determined on an optical microscope using a Malassez chamber after diluting the sample 1:20 in Lazarus liquid. CD34^+^ cells were counted using a dual-platform flow cytometry technique: the blood count was performed on a SFRI H18 Light® cell counter and the CD34^+^ cell count was performed by flow cytometry on a BD FACS CALTO II® cytometer. For allografts with major or bidirectional ABO incompatibility, the hematocrit was measured using an SFRI H18 Light cell counter to estimate the graft red blood cell volume. To assess graft sterility, samples were inoculated into aerobic and anaerobic blood culture bottles.

### Study endpoints and definitions

#### Sex mismatch

Based on donor and recipient sex, allo-HSCT was classified as sex-mismatched specifically when a male recipient received a graft from a female donor.

#### Donor to recipient weight ratio

Bone marrow collection was limited to 20 mL per kilogram of the donor’s body weight, which means that larger donors typically provide larger graft volumes. Because total nucleated cell and CD34^+^ cell doses are calculated based on recipient weight, the final cell dose delivered depends on both graft volume and the relative body size between the donor and recipient. A donor-to-recipient weight ratio greater than 2 was used as a cut-off point to explore this relationship in the analysis.

#### Immunological workup

Before allogeneic transplantation, all patients systematically underwent immunological screening. Anti-HLA antibodies were first assessed, and in cases of positivity, donor-specific antibodies (DSA) were subsequently identified using a Luminex single-antigen bead assay. The clinical team, as an indication for a desensitization cycle, set a threshold of positivity ≥1500 mean fluorescence intensity (MFI). Depending on the treating physician's assessment, this involved one or a combination of the following modalities: administration of an anti-CD20 monoclonal antibody (rituximab), plasmapheresis, and intravenous administration of polyvalent immunoglobulins. In addition, a lymphocyte crossmatch test was performed for every patient to ensure donor–recipient compatibility. This comprehensive immunological evaluation was conducted as part of the standard pre-transplantation workup to minimize the risk of graft rejection and improve engraftment outcomes.

#### Conditioning regimen

Two types of conditioning regimens were used for patients receiving haploidentical allo-HSCT: myeloablative conditioning (MAC) with busulfan as the alkylating agent, and non-myeloablative conditioning (NMAC) based on immunosuppression.

#### Graft-versus-host disease grading and prophylaxis

Diagnosis and classification of acute and chronic GVHD were carried out according to the criteria used by the European Society for Blood and Marrow Transplantation (EBMT). Classification of acute GVHD followed the modified Glucksberg classification [9,10]. Grades II and IV are considered acute GVHD. Classification of chronic GVHD followed the classification of Jagasia et al. [11].

All patients received GVHD prophylaxis with a combination of cyclosporine A (CsA), mycophenolate mofetil (MMF) and post-transplant cyclophosphamide. Cyclophosphamide was administered at a dose of 50 mg/kg on Days +3 and +4 post-transplant. CsA and MMF were given continuously at doses of 1.5 mg/kg every 12 h and 45 mg/kg/day, respectively.

## Statistical analysis

Data, collected from the patient files, were processed using Microsoft Office Excel® 2016. Statistical analysis was carried out using MedCalc® 19.0.4 software. To better reflect the distribution of the data and facilitate their interpretation for statistical analysis, continuous variables were expressed as medians with interquartile ranges [Q1; Q3] and categorical variables were presented as counts (n) and percentage (%). Calculation of the median and the 25th and 75th quartiles was chosen to qualify graft volume and cellular richness of MNC and CD34^+^ cells. Univariate analysis of clinical and transplant-related variables was performed using contingency tables, with crude odds ratios (ORs) and 95% confidence intervals (95% CI) calculated to assess their association with the incidence of acute and chronic GVHD. To further identify independent predictors of GVHD, a multivariate logistic regression model was performed.

## Results

Forty-eight consecutive haploidentical bone marrow allo-HSCT were included in this study with a total of 47 patients (one patient was transplanted twice with two different donors). The characteristics of study population are presented in Table 1.

**Table 1:** Patient and donor characteristics.

Patient	N = 47
Age, years - median (range)	2 (2 months −16 years)
Males / Females - n (%)	22 (46.80)/25 (53.19)
Weight, kg - median (range)	11.6 (4.7–51.9)
Diagnosis	
Hematologic malignancies - n (%)	24 (51.1)
Myelodysplastic syndromes	2 (4.25)
T-cell Acute lymphoblastic leukemia	2 (4.25)
B-cell Acute lymphoblastic leukemia	10 (21.27)
Acute myeloid leukemia	10 (21.27)
Non-malignant disorders	23 (48.9)
Chronic granulomatous disease	3 (6.38)
Aplastic anemia	6 (12.76)
Severe Combined Immunodeficiency	12 (25.53)
Immunodeficiency by defective expression of MHC class II – n (%)	1 (2.12)
Leukocyte adhesion deficiency LFA1 – n (%)	1 (2.12)
Conditioning intensity – n (%)	
Non-myeloablative	4 (8.5)
Myeloablative	43 (91.5)
Donor Specific Antibodies – n (%)	
Negative	41 (87.2)
Positive	6 (12.8)

Donor	N = 48

Age, years - median (range)	38 (9–55)
>18 years old	3 (6)
<18 years old	45 (93.75)
Males / Females - n (%)	30 (62.50)/18 (37.50)
Weight, kg - median (range)	73 (28–115)
Same-sex – n (%)	17 (35.4)
Female to male – n (%)	12 (25)
Male to female – n (%)	19 (39.6)
Donor to recipient relationship – n (%)	
Parent	
Mother	16 (33)
Father	28 (59)
Sibling	
Brother	3 (6)
Sister	1 (2)
ABO identical – n (%)	32 (67)
ABO minor mismatch – n (%)	5 (10)
ABO major mismatch – n (%)	10 (21)
ABO bidirectional mismatch – n (%)	1 (2)

### Graft characteristics

Table 2 shows detailed characteristics of the grafts. A median graft volume of 260.5 mL (IQR: 187.75–388.50) was found. The 25th, 50th and 75th centiles were selected as thresholds to analyze the impact of cell doses on transplant outcomes: these were 3.95 × 10^8^/kg, 7.01 × 10^8^/kg and 9.31 × 10^8^/kg for TNC, and 4.46 × 10^6^/kg, 8.7 × 10^6^/kg and 14.36 × 10^6^/kg for CD34^+^ cells, respectively.

**Table 2:** Graft characteristics.

Prepared graft	N = 48
Volume (mL)	260.50 (187.75– 88.50)
MNC × 10^8^/kg recipient body weight	7.01 (3.95–9.31)
CD34^+^ × 10^6^/kg recipient body weight	8.70 (4.46–14.36)

MNC: Mononuclear cells.

### Univariate analysis of acute and chronic graft-versus-host disease

As shown in Table 3, most factors, including graft cellular content (CD34⁺ and MNC dose), conditioning regimen, underlying hematologic malignancies, donor age, DSA and ABO incompatibility, did not significantly influence the occurrence of acute GVHD. Conversely, donor–recipient sex mismatch emerged as a strong predictor and was significantly associated with an increased risk of acute GVHD (OR = 10.0; 95% CI: 2.26–44.20; p = 0.0024). Regarding chronic GVHD, none of the analyzed variables reached statistical significance, although trends were observed for a higher risk with sex mismatch (OR = 3.4; p = 0.25) and DSA positivity (OR = 2.6; p = 0.44). These findings suggest that, in this cohort, sex mismatch represents the main determinant of acute GVHD, whereas no clear factor was identified for chronic GVHD.

**Table 3:** Univariate analysis of risk factors for acute grade II-IV and chronic graft-versus-host disease occurrence.

Factor	Acute graft-versus-host disease		
	OR	95% CI	p-value
CD34^+^ ≥4.46 × 10^6^/kg	2.1600	0.4027–11.5863	0.3689
MNC ≥3.95 × 10^8^/ kg	0.6481	0.1556–2.6997	0.5514
Conditioning regimen	0.5769	0.05866–5.6740	0.6372
Hematologic malignancies	0.9412	0.2695–3.2866	0.9243
Donor age >18 years old	3.2222	0.1560–66.5458	0.4488
Donor specific antibodies	0.4462	0.04728–4.2099	0.4809
Minor ABO incompatibility	1.1500	0.1803–7.3333	0.8825
Major ABO incompatibility	0.9857	0.2107–4.6113	0.9854
Bidirectional ABO incompatibility	6.7143	0.2521–178.8513	0.2555
Sex mismatch	10.0000	2.2622–44.2046	0.0024
Donor–recipient weight >2	0.7949	0.07550–8.3685	0.8484

	Chronic graft-versus-host disease		

CD34^+^≥4.46 × 10^6^/kg	0.2571	0.03173–2.0840	0.2033
MNC ≥ 3.95 × 10^8^/ kg	0.8824	0.08244–9.4436	0.9176
Conditioning regimen	0.7980	0.03761–16.9329	0.8849
Hematologic malignancies	2.0000	0.1688–23.6963	0.5826
Donor age >18 years old	0.7590	0.03356 - 17.1692	0.8624
Donor specific antibodies	2.6000	0.2250–30.0465	0.4441
Minor ABO incompatibility	2.0000	0.1720–23.2525	0.5797
Major ABO incompatibility	0.4150	0.01975–8.7193	0.5713
Bidirectional ABO incompatibility	2.9048	0.09827–85.8643	0.5371
Sex mismatch	3.4000	0.4235–27.2960	0.2495
Donor–recipient weight >2	1.0000	0.04597–21.7550	1.0000

OR: Odds ratio; 95% CI: 95% confidence interval; MNC: Mononuclear cells.

### Multivariate analysis of risk factors for acute and chronic graft-versus-host disease

The results of the multivariate logistic regression model, performed to identify independent predictors of GVHD, are summarized in Table 4. For acute GVHD, most factors, including graft cellular dose, conditioning regimen, disease type, donor age, DSA, and ABO incompatibility, did not retain statistical significance. However, donor–recipient sex mismatch remained strongly associated with an increased risk of acute grade II–IV GVHD (OR = 17.7; 95% CI: 0.13–297.9; p = 0.0068). In contrast, no variable emerged as an independent risk factor for chronic GVHD in this cohort, as all tested parameters failed to reach statistical significance.

**Table 4:** Multivariate logistic regression model of risk factors for acute grade II-IV and chronic graft-versus-host disease after haploidentical allogeneic stem cell transplantation.

Factor	Acute graft-versus-host disease			
	Coefficient	OR	95% CI	p-value
CD34^+^ ≥4.46 × 10^6^/kg	2.39458	10.9636	[0.5626–213.63]	0.1140
MNC ≥3.95 × 10^8^/kg	0.028126	1.0285	[0.0836- 12.66]	0.9825
Conditioning regimen	2.2110	0.5578	[0.0243–12.824]	0.7152
Hematologic malignancies	0.31894	1.3757	[0.2060–9.1855]	0.7420
Donor age >18 years old	17.27854	31.9e+006	-	0.9987
Donor specific antibodies	−1.38597	0.2501	[0.0103–6.0538]	0.3940
ABO compatibility	0.29541	1.3437	[2.2110–8.4759]	0.7532
Sex mismatch	2.87369	17.7022	[0.1326–297.88]	0.0068
Donor–recipient weight >2	−17.91529	0.0000	-	0.9985

	Chronic graft-versus-host disease			

CD34^+^ ≥4.46 × 10^6^/kg	−20.27110	1.57E-009	-	0.9980
CMN ≥3.95 × 10^8^/ kg	0.030754	1.0312	[0.0280–38.02]	0.9867
Conditioning regimen	−40.15163	3.65E-018	-	0.9990
Hematologic malignancies	0.78240	2.1867	[0.0595–80.31]	0.6704
Donor age >18 years old	0.60455	1.8304	-	1.0000
Donor specific antibodies	21.84481	3.07E+009	-	0.9990
ABO compatibility	−0.13319	0.8753	-	1.0000
Sex mismatch	21.22466	1.65E+009	-	0.9979
Donor–recipient weight >2	−17.91529	2.46E-018	-	0.9989

OR: Odds ratio; 95% CI: 95% confidence interval; MNC: Mononuclear cells.

## Discussion

In this study, univariate analysis examining the occurrence of acute GVHD identified one statistically significant factor associated with an increased risk of acute GVHD: donor–recipient sex mismatch. Furthermore, in multivariate analysis, gender mismatch continues to be a statistically significant factor, while some factors did not reach statistical significance in the analysis. This may reflect either a limited role in the development of acute GVHD or constraints related to sample size. These findings do not rule out the potential relevance of some other factors and future studies with larger patient populations could provide more definitive insights.

Several studies have demonstrated that recipient minor histocompatibility antigens are targets of donor T cells, playing a central role in mediating both GVHD and the graft-versus-leukemia (GVL) effect. These minor H antigens originate from cellular proteins derived from polymorphic genes, which differ between donor and recipient. The corresponding peptides are then presented to CD8^+^ and CD4^+^ T lymphocytes via class 1 and 2 major histocompatibility complexes, respectively. During allo-HSCT from a female donor to a male recipient, donor T cells recognizing minor H antigens specific for the recipient Y chromosome genes may be present, resulting in both GVHD and GVL effects. The Y chromosome genes encoding these H antigens have already been identified and their roles in GVHD and GVL are currently under investigation. Bross et al. published the results of a study involving 136 patients. in which 71% of patients who received an opposite-sex donor transplant developed grade II-IV acute GVHD [12]. Similarly. Randolph et al. conducted a retrospective analysis of 3238 patients who underwent allo-HSCT with a genoidentical related donor. After receiving myeloablative conditioning a statistically significant OR was observed in male recipients who received female donor transplants for both acute grade II-IV GVHD and chronic extensive GVHD [13,14]. Since previous pregnancies or transfusions may influence the likelihood of donor-derived cells causing GVHD, Flowers et al. considered this variable in their study. Of the 136 genoidentical bone marrow donors, 30 were multiparous women, 30 nulliparous women and 76 were men. The cumulative incidence of grade II-IV acute GVHD was 57% for patients with multiparous female donors, 21% for those with nulliparous female donors and 46% for those with male donors. Multivariate analysis of the data confirmed that the risk of grade II IV acute GVHD was significantly higher in patients who received multiparous donor transplants compared with those who received nulliparous donor transplants (RR = 2.5; p = 0.02) [15].

The present study revealed no statistically significant factors associated with the development of chronic GVHD, likely reflecting limited sample size and statistical power. However, the complex and multifactorial nature of chronic GVHD likely also contributes to the lack of clear predictive patterns in this cohort.

Among the factors influencing the outcome of allo-HSCT, GVHD prophylaxis plays a key role particularly in preventing this dreaded complication. This field has seen many advances and remains at the heart of numerous scientific investigations and clinical trials exploring various protocols to optimize GVHD prevention and treatment. In the current study, GVHD prophylaxis was performed with the triad: CsA, MMF and post-transplant cyclophosphamide. In recent years, several strategies have been developed and refined to improve the results of haploidentical allo-HSCT, with a particular focus on reducing GVHD while maintaining a robust anti-leukemic effect for hematological malignancies. These innovative approaches include the use of post-transplant cyclophosphamide, as well as other methods, which continue to be the subject of in-depth study and clinical evaluation. The Baltimore group first described the use of post-transplant cyclophosphamide for haploidentical allogeneic HSCT. Luznik et al. proposed a novel approach based on the use of cyclophosphamide post-transplant, combined with non-myeloablative conditioning and the use of bone marrow as the cell source. Administration of high doses of cyclophosphamide equal to 50 mg/kg/day on the third and fourth days after haploidentical allo-HSCT enables selective elimination of alloreactive T cells, while preserving regulatory T cells and hematopoietic stem cells. Brunstein et al. and Luznik et al. demonstrated low rates of GVHD (6% for grade III-IV acute GVHD and 13% for moderate or severe chronic GVHD), as well as a non-relapse mortality rate of <15% [16,17]. The low toxicity of this strategy, combined with the non-myeloablative conditioning proposed by the Baltimore team, makes it a particularly attractive option for elderly patients, who are generally at greater risk of complications. Several studies have confirmed this low toxicity in this population, and single-center retrospective comparisons and registry data have revealed survival rates comparable to those of HLA-matched transplants [[18], [19], [20]]. However, due to the non-myeloablative nature of conditioning, this approach is limited by high relapse rates of up to 50% [16,17]. Several studies have shown that higher-intensity conditioning improves disease control, reducing relapse rates to between 20% and 40% [[21], [22], [23]]. Ultimately, there are many arguments in favor of this strategy: it is simple to implement, inexpensive and associated with low rates of graft failure, GVHD, infection and mortality not related to relapse [24]. Current research is focused on understanding the mechanisms of immune reconstitution after haploidentical allo-HSCT with high doses of post-transplant cyclophosphamide. Mastering these mechanisms would make it possible to exploit the beneficial alloreactivity linked to HLA incompatibility between donor and recipient. Several models also suggest that it is possible to specifically modulate the activity of immune cells, such as T or NK lymphocytes, to achieve an anti-tumor effect without the complications associated with GVHD. This approach redefines allogeneic immunotherapy. The establishment of a partially compatible allogeneic immune system, under conditions of reduced toxicity can provide the basis for targeted anti-tumor immune interventions. Currently, in vivo T-cell depletion strategies are tending to supplant those based on in vitro depletion, as evidenced by analysis of the European Society for Blood and Marrow Transplantation registry. The majority of centers specializing in haploidentical transplants have opted for the post-transplant cyclophosphamide approach, enabling the use of reduced-intensity conditioning and, consequently, the treatment of older patients [25,26].

Since 2010, the number of haploidentical HSC transplants has increased substantially worldwide gradually overtaking placental blood transplants and unrelated donor transplants with 9/10 compatibility, reversing the trend observed since 2012 in France [27]. In Europe, the use of haploidentical family donors is growing by around 25% each year [28]. Initially reserved for patients requiring allo-HSCT and for whom no compatible donor was available, this approach has rapidly become widespread. The encouraging results, the near-certainty of finding a donor within the family, the reduced cost and the shorter time it takes to identify a donor have all contributed to its growing popularity. Today, the place of this strategy within the therapeutic framework of allografts, as well as its position in the hierarchy of donor choices, is the subject of much debate within the scientific community.

## Conclusions

In this cohort, donor–recipient sex mismatch was the main risk factor for acute GVHD, while no clear predictor was identified for chronic GVHD. A broader analysis of the patient cohort, together with an increase in sample size, will enable us to refine the predictive models and reinforce the robustness of the conclusions drawn. The aim will also be to better identify the impact of certain hitherto underestimated factors, and to continue exploring the complex interactions between different variables in order to optimize haploidentical transplant protocols. Continuing this work could open up new prospects for personalizing treatments and improving outcomes for transplant patients, while reducing the risk of complications.

## CRediT authorship contribution statement

Meriem Lameri: Writing – Original Draft, Methodology, Investigation, Data Curation, Formal analysis, Conceptualization, Visualization. Monia Ben Khaled: Methodology, Investigation, Resources. Eya Ghazouani: Data Curation, Formal analysis. Ilhem Ben Fraj: Methodology, Investigation, Resources. Mohamed Hichem Sellami: Formal analysis, Superviosion. Wafa Aissa: Investigation. Yesmine Boughzala: Investigation. Houda Kaabi: Resources, Investigation. Slama Hmida: Resources, Supervision. Monia Ouederni: Conceptualization, Methodology, Investigation, Resources, Writing – Review & Editing, Supervision. Manel Chaabane: Conceptualization, Methodology, Writing – Review & Editing, Resources, Project administration, Validation, Supervision.

## Ethical approval

There are no ethical issues.

## Source of funding

This study did not receive any specific grant from funding agencies.

## Data availability

The data that support the findings of this study are available from the corresponding author upon reasonable request.

## Conflicts of interest

The authors declare that they have no known competing financial interests or personal relationships that could have appeared to influence the work reported in this paper.
